# Radiofrequency as the New Opportunity in Treating Overactive Bladder and Urge Urinary Incontinence—A Single-Arm Pilot Study

**DOI:** 10.3390/medicina60020197

**Published:** 2024-01-24

**Authors:** Damir Franić, Maja Franić Ivanišević, Ivan Verdenik

**Affiliations:** 1 Ginekologija Dr. Franić d.o.o., 3250 Rogaška Slatina, Slovenia; 2 Medical Faculty, University of Maribor, 2000 Maribor, Slovenia; 3 Obstetric and Gynecology Unit, Health Centre Slovenske Konjice, 3210 Slovenske Konjice, Slovenia; dr.ivanisevic@gmail.com; 4 Research Unit, University Gynecological Clinic Ljubljana, 1000 Ljubljana, Slovenia; ivan.verdenik@guest.arnes.si

**Keywords:** overactive bladder, urge incontinence, radiofrequency, treatment

## Abstract

*Background and Objectives*: Until now, overactive bladder (OAB) with or without urge urinary incontinence (UUI) has been treated mainly in two ways: with behavioral methods and patient education, or using antimuscarinic drugs and/or beta-3 adrenergic receptor agonists. Unfortunately, these drugs may cause side effects in some women or are insufficiently effective, so patients abandon them. Therefore, in this pilot study, radiofrequency was evaluated as a new option in the treatment of OAB and UUI. *Materials and Methods*: Nineteen patients were enrolled in this pilot study using radiofrequency (RF), where the level of OAB and UUI was assessed using the validated ICIQ-OAB questionnaire. RF was applied four times for 20 min, once a week. Two weeks after treatment, the level of OAB and UUI was reassessed and processed statistically and the treatment effect evaluated. *Results*: Using the ICIQ-OAB, the severity of OAB and UUI was assessed: 0–3 mild symptoms; 4–7 moderate symptoms; 8–11 severe symptoms; 12–16 very severe symptoms. Before treatment, 10.5% of patients had mild symptoms, 21.1% moderate symptoms, 63.2% severe symptoms and 5.3% very severe symptoms. After treatment, 42.9% had mild symptoms, 50% moderate symptoms and 7% severe OAB and UUI symptoms. All four main symptoms—frequency, nocturia, urgency and incontinence—decreased statistically significantly, with the best results being found in urgency (*p* = 0.002). *Conclusions*: Based on this pilot study, RF seems a very promising method in the treatment of OAB and UUI. To extend our initial findings, it is necessary to perform a prospective, randomized and placebo-controlled study in order to obtain reliable results and to determine for how long one set of treatment maintains the results obtained immediately after the end of that treatment. In this way, we may determine how often the treatment needs to be repeated, if necessary, and when.

## 1. Introduction

Our conceptions and knowledge of OAB have undergone many changes over the past 25 years. In the past, bladder oversensitivity and urge incontinence were considered two separate entities and were pharmacologically treated only with first-generation antimuscarinics such as propantheline and oxybutynin [1]. At the beginning of the 21st century, a new concept was established that combines four connected symptoms, which today is known as OAB [1]. As defined by the International Incontinence Society (ICS), OAB features urinary urgency as a leading symptom with or without UUI, often combined with urinary frequency and nocturia. The primary urodynamic disorder that characterizes OAB is detrusor hyperactivity. Urination is slow, often interrupted, in drops and there is straining while waiting for the stream [2,3]. The prevalence of OAB worldwide varies widely from 1% to 38.8% [4].

There are four levels in the treatment of OAB: first level—behavioral treatment (BT) with lifestyle changes and patient education; second level—pharmacological treatment with (a) antimuscarinics and (b) beta-3 agonists; third level—the use of botulinus toxin, peripheral stimulation n. tibialis (PSNT) and sacral neuromodulation (SNM) [2,3]; and fourth level—the use of a permanent catheter, plastic bladder augmentation and diversion of urination. In practice, the first two levels are the most established—behavioral treatment with patient education and lifestyle changes, and pharmacological treatment. The third and fourth levels of treatment are used only in the most severe cases in which no other treatment has produced results [2,3]. The use of antimuscarinics and/or beta-3 agonists is currently the most established treatment for OAB [5,6,7,8]. Regardless of the effectiveness and development of antimuscarinics, it should be emphasized that there is a significant increase in the possibility of developing dementia for the elderly who use drugs with antimuscarinic properties in their daily life, in addition to other general side effects typical of antimuscarinics [9,10]. The combination of antimuscarinics with beta-3 agonists shows good synergistic action and improvement in the quality of life in patients with OAB [11,12].

We are obligated to remember our oath—“primum nil nocere”—which asks us to find solutions that help the patient as much as possible with the fewest side effects. In this regard, the use of radiofrequency (RF) in the treatment of OAB and UUI presents a new possibility to help patients who, either due to the side effects of drugs and/or their ineffectiveness, cannot use pharmacological treatment; by applying RF, we may be able to improve their quality of life, as was investigated in this study.

## 2. Methods

A prospective, single-arm pilot study was conducted from January 2021 to September 2021, in which we included 19 women with symptoms of OAB and UUI, as confirmed with a completed, validated ICIQ-OAB questionnaire, both before radiofrequency treatment and again 2 weeks after the treatment. All patients gave informed consent before entering the study. At the same time as completing the questionnaire, all patients provided a urine sample, which we analyzed with a urine culture to exclude possible urinary infection. This study was approved by the National Ethics Committee of the Republic of Slovenia (No. 0120-184-2023).

### 2.1. Criteria for Inclusion in This Study

Women who had clear signs of OAB and who did not use drugs for its treatment (antimuscarinics/beta-3 agonists) were included.

Women who had taken drugs for the treatment of OAB in the past but stopped (side effects, ineffectiveness), with at least 3 months since their last treatment, were also included in this study.

### 2.2. Exclusion Criteria for This Study

Exclusion criteria included an inserted heart pacemaker, unexplained bleeding from the vagina, inflammation of the vagina and/or uterus or uterine appendages, bacterial or viral infection, impaired immune system, scleroderma, radiation treatment, or burns in the treatment area. Women with stress urinary incontinence (SUI) or mixed urinary incontinence were not included in this study.

### 2.3. Radiofrequency in the Treatment of Urinary Incontinence

In this study, the radiofrequency device Capenergy model C 100, with an upgrade to the urogynecological model C 500, was used. This device has a 1-channel capacitive and resistive probe for individual use. The mechanism of action is electrotherapy with the help of high-frequency electricity (TECAR). The high-power TECAR therapy devices create an electric field in the tissue that causes the molecular movement of the charged particles, generating heat [13].

The use of electromagnetic energy in the organism causes biological and physiological reactions. Since the organism is a conductor of the second level, it consists of a large amount of water with an infinite amount of ions dissolved in it. The application of electromagnetic energy to the organism means the acceleration of metabolic responses at various levels, which are used for therapeutic purposes. TECAR evolution generates energy from 0.8 to 1.2 MHz, which is used for the treatment. The mechanism of action is the use of high-frequency electricity to prompt heat gain in the body tissues as a result of the resistance those tissues offer to the flowing current.

**Figure 1:**
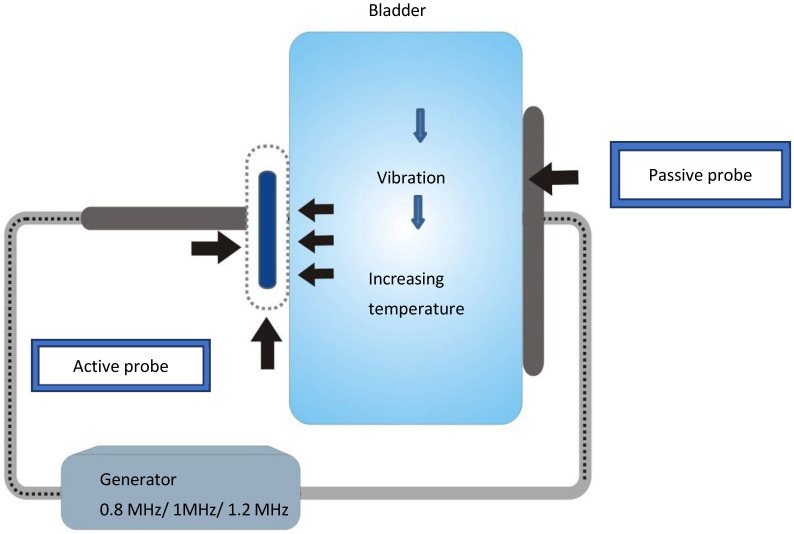
Mechanism of application of high-frequency energy to the urinary bladder area.

The patients were lying in the lithotomy position with stretched lower extremities. Probes were placed on the lower part of the abdomen in the area of the bladder (active probe) and on the lumbar spine (passive probe), as shown in Figure 1. A capacitive probe in the “free treatment”/“power control” program was used, where high-frequency electricity was released to the bladder for 20 min with a frequency of 1.0 MHz and energy limited to that which could produce a maximum temperature of 41 °C. Initially, a maximum energy of 75% and a frequency of 0.8 MHz were applied, which were reduced depending on the amount of heat that was pleasant for the patient. Then, the power was reduced to 50% and the frequency increased to 1.0 MHz, which resulted in moderate energy absorption. The procedure with this level of energy was continued until the end, and the patients absorbed 18–19 kJ of energy on average.

Frequency is the wavelength of the oscillation. Different frequencies are used in diathermy. As tissue absorption increases with frequency, it is often assumed that lower frequencies result in better transfer efficiency. It has been proposed that a frequency of 1 MHz manages to overcome the resistance of the cell membrane and produce intracellular effects [13]. One of the fundamental components of electromagnetic fields is power, or energy. Electromagnetic waves provide energy to a system by virtue of their electric and magnetic fields. The greater the strength of the electric and magnetic fields, the more work they can do and the greater the energy carried by the electromagnetic waves [13].

Such energy desensitizes the bladder and therefore increases its capacity, increasing blood flow and reducing the formation of free radicals, which together improve the symptoms of OAB and UUI.

Furthermore, the finding of an accompanying increase in the production of both epithelial and vascular growth factors, which improve healing and produce angiogenesis, increasing blood circulation, has added a new perspective to this treatment [13].

Beyond that, the other mechanism of radiofrequency with high power in treating OAB and UUI is a diathermic process generated by the radiation of an electromagnetic spectrum, resulting in an immediate retraction of existing collagen and the subsequent activation of fibroblasts, causing non-ablative neocollagenesis. Radiofrequency waves serving this purpose can reach a sufficient depth to induce collagen production in the whole urethra, meaning these mechanisms can improve the symptoms of OAB and UUI [14].

**Figure 2:**
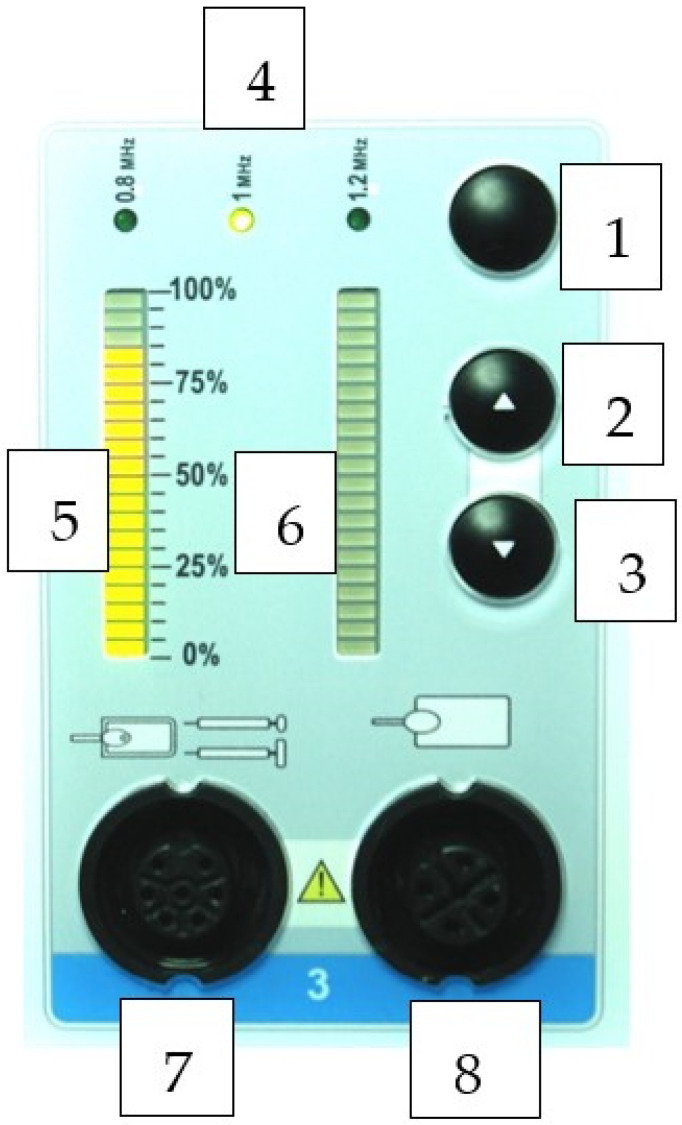
Front panel of the RF machine for adjusting the energy, frequency and the absorption of the energy. CHANNEL: 1—Frequency selection button; 2—Temperature or power increase button; 3—Temperature or power decrease button; 4—Frequency indicator: 0.8 MHz, 1.0 MHz 1.2 MHz; 5—Luminary indicator of the selected power percentage; 6—Qualitative luminary indicator of the immediately delivered power; 7—Active accessory connector; 8—Passive plate connector. Reproduced with permission (User Manual DIATHERMY EQUIPMENT CAPENERGY FAMILY MODELS C 50, C 100, C 200 C 300, C 400, C 500).

The RF application protocol in this study was 1× weekly/20 min/4 weeks. Two weeks after the completion of treatment, the patients filled out the questionnaire again, from which we evaluated the effect of the treatment. The front panel of the RF machine is demonstrated in Figure 2.

### 2.4. Statistics

A comparison of the severity of symptoms before and after the therapy was carried out using the Wilcoxon signed-rank test for paired samples. IBM SPSS Statistics v27 was used for our calculations. Values of *p* < 0.05 were considered significant.

## 3. Results

The demographic characteristics of the subjects are given in Table 1.

Based on the validated ICIQ-OAB questionnaire, which takes into account four main symptoms, namely frequency of urination, nocturia, urgency and incontinence, the levels of OAB and UUI on the basis of the total sum of maximum symptomatology were classified into the following categories (Table 2):

- Mild symptoms: 0–3;
- Moderate symptoms: 4–7;
- Severe symptoms: 8–11;
- Very severe symptoms: 12–16.

Five patients did not show up for the follow-up after the treatment, so we did not include them in the assessment of symptomatology after the treatment. Three of them were completely satisfied with the procedure, but they did not come in to the office to complete the ICIQ-OAB questionnaire, while two of them did not come because they were not satisfied with the procedure. We received feedback from these patients by phone.

**Figure 3:**
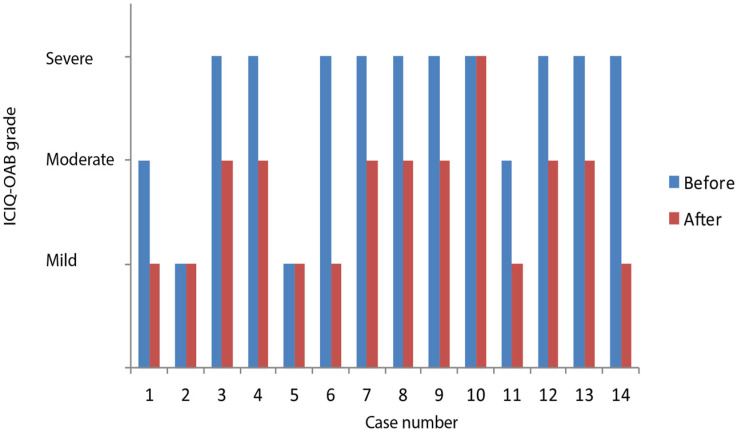
Results before and after treatment according to symptomatology in each patient.

Of those we included in our assessment, there was no difference in symptomatology after the RF procedure in patients 2 and 5 (Figure 3). For all other patients, a significant decrease in OAB symptomatology was shown.

**Figure 4:**
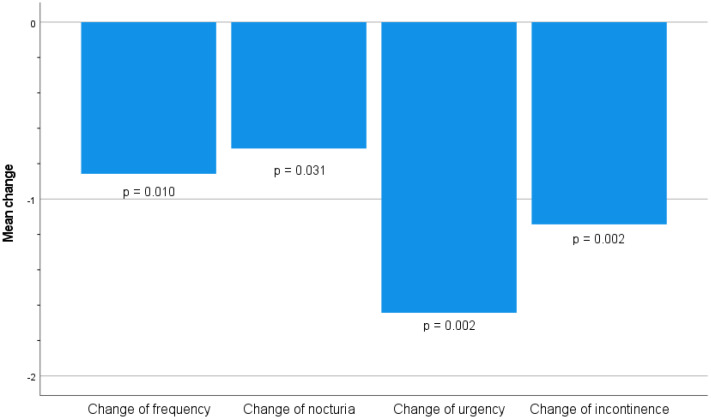
Statistical characteristics of changes in symptomatology “before” and “after”.

Within the statistically significant decrease in overall symptomatology after the treatment, it should be emphasized that the biggest difference was observed in the symptom of urgency (Figure 4).

## 4. Discussion

The main approach for treating OAB and UUI is pharmacological, using antimuscarinics and beta-3 agonists either alone or in combination. As a result of their use, OAB is creating a substantial economic burden in the USA, with an estimated cost of USD 86 billion in 2020 [15]. Furthermore, the use of antimuscarinics in the elderly population causes many side effects, from those typical of antimuscarinics (dry mouth 30%; blurred vision 6%; constipation 8%) [16] to cognitive failure [10,17]. There are five muscarinic receptors (M1–5), of which M2 and M3 are present in the bladder, and M1 and M2 are responsible for cognitive function [18]. In addition to other drugs used by the elderly that also affect M1 and M2 receptors in the brain, the use of antimuscarinics can have a synergistic negative effect on cognitive function.

Unlike antimuscarinics, mirabegron, as the main representative of beta-3 agonists, as well as the newer representative vibegron, did not show any difference in the effect on dementia in placebo controlled studies [19,20]. On the other hand, mirabegron causes hypertension (10%), nasopharyngitis (4.1%) and very rarely dry mouth as side effects [21]. Therefore, as hypertension is present in the elderly, in those with unordered hypertension, antimuscarinics are preferred, but in those patients over 65 years of age, cognitive function monitoring must be performed. Beyond this, studies have added further support to the idea that it matters which antimuscarinic is used. Studies using solifenacin vs. a placebo and oxybutynin vs. a placebo showed that with solifenacin, there were no effects on cognitive function, while with oxybutynin, a significant decline in cognitive function was determined [22]. Similar affirmative conclusions regarding cognition were also found for darifenacin, fesoterodine and tolterodine [23,24,25]. The prescribing of both drugs for the treatment of OAB and UUI must follow considerations of the quality of life. In the context of these treatment complications, efforts to use RF in the treatment of OAB and UUI mark the first attempt to find a possible alternative to pharmacological treatment.

In the treatment of urinary incontinence, numerous studies have described the effectiveness of either a CO_2_ laser or ER:YAG laser in the treatment of stress urinary incontinence, and in some cases, also mixed urinary incontinence [26,27]. The mechanism of the laser is to act on the neocollagenesis of the vagina, taking an approach that is exclusively vaginal.

Alternatively, RF may act on collagen, with its diathermic effect causing collagen denaturation. As the temperature increases, fibroblasts are activated, resulting in neocollagenesis, neoelastogenesis and tissue remodeling [14,28]. The mechanism of RF action on collagen activation is different from laser action, and we tested it in the treatment of OAB and UUI. The resistance created by the organs triggers the healing effect of RF on the tissues through better blood circulation and the reduction of free radicals, and in this way, leads to the desensitization of the urinary bladder, which ultimately results in an increased bladder capacity and a consequent reduction in symptomatology.

Nevertheless, there are currently no studies published on the treatment of OAB and UUI with RF in the peer-reviewed literature. We may only refer to some studies that have been published on the use of RF in the treatment of SUI, namely a 12-month study using cryo-cooled monopolar RF, which did not show statistically significant changes at follow-ups of 1, 4, 6 and 12 months [29]. VIVEVE technology was applied, whose use in women with vaginal laxity and sexual dysfunction consists of 110 pulses of 90 J/cm^2^ at the introitus of the vaginal canal. Average statistical significance was achieved (*p* = 0.02) but the individual differences were large, and paired with the insufficient number of patients, that led to a dubious conclusion. Another study [30] was performed using the non-ablative radiofrequency device Spectra G2—Tonederm^®^ on the urethral meatus. The researchers applied a PAD test at the end of treatment and after 1, 2 and 3 months of follow-up. In the final PAD test, 70% of subjects showed a reduction and 30% a worsening of urinary loss.

Elsewhere in the literature, in a review article and meta-analysis of minimally invasive interventions in the treatment of OAB, only 14 relevant studies were identified from the database and included in the analysis [31]. In those studies, statistically significant results were achieved in transcutaneous tibial nerve stimulation (TTNS), pelvic floor muscle training (PFMT) and behavioral therapy (BT).

In a further investigation, acupuncture did not give significant results in the treatment of OAB [32].

In our study, statistically significant results 2 weeks after the completed treatment were achieved for all four symptoms of OAB and UUI. The best results were achieved for urgency, which is the basic symptom that confirms OAB.

Adding to those promising findings are certain key benefits of RF treatment, such as freedom from side effects if precautions are taken to prevent hyperthermia and the possibility of burns at the site of the active probe on the abdomen.

However, this is the first attempt to use the RF method in the treatment of OAB and UUI, and only a small number of patients were included in this pilot study. Furthermore, we did not have a control group, which presents a key limitation to this study.

## 5. Conclusions

It should be emphasized that this is the first demonstration of the treatment of OAB and UUI with RF. In the future, it is necessary to conduct a prospective, placebo-controlled and randomized study in order to determine definitively how RF affects OAB and UUI. Above all, it is important to monitor the “follow-up” to determine how much the initial treatment maintains this effect and, based on this, to create a treatment protocol. Based on the findings reported herein, in which the energy applied to the bladder during the treatment was at a level lower than any that would produce nerve injury in the pelvic area, we propose that it is safe to proceed with such studies.

## Figures and Tables

**Table 1:** Demographics.

	Mean ± SD or *n* (%)	Min–Max
Age	57.9 ± 13.0	38–71
Age categories		
<50 years	5 (26.3%)	
50–59 years	5 (26.3%)	
≥60 years	9 (47.4%)	
BMI	28.5 ± 3.9	21–35
BMI categories		
BMI < 25	2 (10.5%)	
BMI 25–29.99	9 (47.4%)	
BMI ≥ 30	8 (42.1%)	
Maternal history—parity	2.37 ± 1.21	1–6

**Table 2:** Classification of female patients according to the degree of symptomatology.

	Grade before the Therapy		Grade after the Therapy	
	*n* = 19	%	*n* = 14	%
Mild	2	10.5	6	42.9
Moderate	4	21.1	7	50.0
Severe	12	63.2	1	7.1
Very severe	1	5.3	0	0.0
Missing	0		5	
Total	19	100.0	19	100.0

## Data Availability

Data are contained within the article.
